# Challenges for Australia's Bio/Nanopharma Policies: trade deals, public goods and reference pricing in sustainable industrial renewal

**DOI:** 10.1186/1743-8462-4-9

**Published:** 2007-06-01

**Authors:** Thomas A Faunce

**Affiliations:** 1 College of Law and Medical School, Australian National University, Canberra, Australia

## Abstract

Industrial renewal in the bio/nanopharma sector is important for the long term strength of the Australian economy and for the health of its citizens. A variety of factors, however, may have caused inadequate attention to focus on systematically promoting domestic generic and small biotechnology manufacturers in Australian health policy.

Despite recent clarifications of 'springboarding' capacity in intellectual property legislation, federal government requirements for specific generic price reductions on market entry and the potential erosion of reference pricing through new F1 and F2 categories for the purposes of Pharmaceutical Benefits Scheme (PBS) assessments, do not appear to be coherently designed to sustainably position this industry sector in 'biologics,' nanotherapeutics and pharmacogenetics.

There also appears to have been little attention paid in this context to policies fostering industry sustainability and public affordability (as encouraged by the National Medicines Policy). One notable example includes that failure to consider facilitating mutual exchanges on regulatory assessment of health technology safety and cost-effectiveness (including reference pricing) in the context of ongoing free trade negotiations between Australia and China (the latter soon to possess the world's largest generic pharmaceutical manufacturing capacity). The importance of a thriving Australian domestic generic pharmaceutical and bio/nano tech industry in terms of biosecurity, similarly appears to have been given insufficient policy attention.

Reasons for such policy oversights may relate to increasing interrelationships between generic and 'brand-name' manufacturers and the scale of investment required for the Australian generics and bio/nano technology sector to be a significant driver of local production. It might also result from singularly effective lobbying pressure exerted by Medicines Australia, the 'brand-name' pharmaceutical industry association, utilising controversial interpretations of reward of pharmaceutical 'innovation' provisions in the Australia-US Free Trade Agreement (AUSFTA) through the policy-development mechanisms of the AUSFTA Medicines Working Group and most recently an Innovative Medicines Working Group with the Department of Health and Ageing. This paper critically analyses such arguments in the context of emerging challenges for sustainable industrial renewal in Australia's bio/nanopharma sector.

## Background

Australian pharmaceutical exports were A$1.77 billion in 1999–2000 and approximately 14,000 people were employed in the industry. By 2003, Australia was exporting $1.96 billion in pharmaceutical products, but importing $4.4 billion (of which 45% derived from firms of US corporate nationality). None of the top ten pharmaceutical firms (by sales volume) was Australian [1]. It is unlikely in the foreseeable future, chiefly because of the scale of investment required, that large scale Australian-owned 'innovative' or research-oriented pharmaceutical companies will become a sustainable proposition.

This sanguine conclusion and impending patent expiries over high sales revenue ('blockbuster') medicines, should have spurred Australian government policies for systematic industrial renewal, paying careful attention to supporting the generic pharmaceutical sector (understood as the conditions for supply of generic medicines in Australia). Such reforms could reasonably be expected to have positioned that sector favourably in relation to challenges like those concerning biologic products, nanotechnology and pharmacogenetics. As this article attempts to show, such systematic regulatory reforms have not eventuated. Instead, the Australian generics industry has reacted with dismay and anger at many recent government policies, developed after relatively low levels of consultation and with little apparent appreciation of the long term challenges for medicines policy in Australia [2]. This article complements that of Lofgren in this series, by exploring the factors influencing, and the consequences of, this policy oversight regarding Australia's emerging biotech/nanotech sector, as well as the importance in that context of reference pricing as a means of promoting markets genuinely competitive for public health outcomes as well as sustainable profits.

### Australia's Generic Pharmaceuticals Policy in a Global Context

Worldwide, generic pharmaceutical manufacturers comprise a large segment of the global pharmaceutical industry, and are collectively expanding at a faster rate than the so-called 'innovative' or 'brand name' sector, as a consequence of systematic regulatory encouragement, mergers and acquisitions, as well as the growth of a new market for 'biosimilars' [3]. Generics sales (in the top eight national markets) in 2005 were about US$55 billion, which represents about one tenth of the total global prescription drug market [4]. A UK-based business forecaster has predicted the Australian generic pharmaceuticals market, with appropriate regulatory support, should have doubled in value to $2.4 billion a year by 2009 on the back of three per cent rise in market share from the current estimate of 12.8 per cent.[5] As we shall see, for a variety of structural and regulatory reasons, such estimates now likely to be more conservatively revised.

Under the Pharmaceutical Benefits Scheme (PBS) cost-effectiveness reimbursement system, as it operated between the late 1990's and 2004, Australian generic pharmaceutical firms competed not on PBS price, but for deals with retail pharmacists (by offering convenient supply arrangements and, most significantly, large discounts, in the order of 30% or more). They also benefited (as did the Australian public and Federal government) from the process of reference pricing and cost-effectiveness assessments involved in the Australian PBS listing process. This ensured the public relatively favourable prices for 'brand-name' medicines, but permitted slightly higher prices for generics, compared to the US and some other developed countries [6]. Claims that the PBS prevented generic suppliers engaging in price competition were exaggerated and misleading [7]. The PBS has unquestionable democratic legitimacy. It is one of the few pieces of public policy in Australia that has been approved in a Constitutional referendum by a majority of citizens in a majority of States. It has survived challenges to its implementing legislation in the High Court of Australia and been improved by a series of federal governments over more than fifty years of intense health policy debate [8].

The core regulatory component of the PBS system is section 101 (3A&B) of the *National Health Act 1953 (Cth)*. This, in broad terms, requires that pharmacoeconomic experts on the Pharmaceutical Benefits Advisory Committee (PBAC), can only recommend PBS listing of a pharmaceutical submitted by its manufacturer after a positive determination of its cost-effectiveness in relation to alternative therapies (whether or not involving drugs). If the submitted product is proven to be substantially more costly than such comparitors, then a significant improvement in efficacy or reduction in toxicity has to be established to justify listing. This provision provides the legislative basis for reference pricing under the Australian PBS.

Reference pricing is a central component of the basic architecture of the PBS system [9]. When cost-effectiveness analysis has determined that a new patented drug seeking PBS listing provides no greater efficacy or safety than is appropriate comparitor, then an exercise of cost-minimisation begins in which its price is 'referenced' down to that of the comparitor. Reference pricing thus is an evidence-based mechanims for accountably and transparently valuing pharmaceutical innovation on grounds of objectively demonstrated therapeutic significance [10]. Prices of all drugs in such a group are tied to that of the lowest, or in some cases the average, price [11]. This does not necessarily mean that the reference price becomes the market price for all drugs in the same therapeutic class, rather the reference price becomes a benchmark [12]. Manufacturers can set prices higher than the reference, but in doing so they need to genuinely compete in the open market against equivalent lower priced medicines. This is one reason why it is inaccurate to say that reference pricing inhibits competition on price. The resultant expert recommendation may allow the creation of either positive or negative lists for government reimbursement of pharmaceutical prices [13].

Between 1990 and 2004, a succession of Australian governments funded a variety of regulatory initiatives, to obtain greater public benefit from the PBS system, pharmaceutical R&D and the generic pharmaceuticals sector. Reference pricing and the capacity it gave government reimbursement to value innovation scientifically against criteria of objectively demonstrated therapeutic significance, was central to capacity of these policy initiatives to fulfil the core elements of the Australian National Medicines Policy. These reflected a fair balance of all stakeholder concerns:

1. timely access to the medicines that Australians need, at a cost individuals and the community can afford;

2. medicines meeting appropriate standards of quality, safety and efficacy;

3. quality use of medicines; and

4. maintaining a responsible and viable medicines industry [14].

One Australian pharmaceutical industry initiative involved the minimum pricing policy, introduced in December 1990. This encouraged patients to switch from innovator brands to corresponding generic products. The effect, however, was marginal, since pharmacists could only dispense the brand prescribed by the doctor, and the average surcharge or brand premium was only about $1 per prescription. Brand substitution by pharmacists was introduced on 1 December 1994, when generic medicines constituted only 2% of PBS expenditure. This policy allowed supply of less-expensive generic medicines at the request of the patient, regardless of which brand the doctor had prescribed. In February 1998, the government introduced the therapeutic group premiums scheme (TGPs). Its objective was to introduce greater competition into the pricing of medicines judged to have an equivalent therapeutic effect, even though they were not identical chemical compounds. The availability of cheaper generics was then supposed to have flow-on positive effects in decreasing PBS reimbursement of all products in such groups (despite the extent of brand premiums).

On 29 May 2001, the then Minister of Industry, Tourism and Resources announced a Pharmaceuticals Industry Action Agenda with an Implementation Group under the Chairmanship of Dr Graeme Blackman. Its key policy recommendations were to "promote increased investment and exports of pharmaceuticals goods and services" (action 2); "identify opportunities and facilitate growth in the export of pharmaceuticals industry" (action 7) "promote two-way movement between industry and academia" (action 11) and "align industry activity with the National Innovation Awareness Strategy" (action 14) [15].

As part of this Action Agenda, and following on from similar programs dating from the late 1980s, the Department of Industry, Tourism and Resources between 1999 and 2004 operated the $300 million Pharmaceutical Industry Investment Program which rewarded manufacturers undertaking research and development in Australia. This program channelled support to nine companies, including one generics firm, FH Faulding & Co Limited (subsequently Mayne Pharma) [16]. It was replaced from 1 July 2004 by the Pharmaceuticals Partnerships Program worth $150 million over five years.

These policies focused on subsidising research and development and not on making the types of structural and regulatory changes that would support the sustainability of a sustainable Australian generic pharmaceutical industry linked by incentives to nano/biotechology companies and increased science education infrastructure. Crucial to such sustainability is a system of high rewards for genuine innovation objectively demonstrated by expert comparison of outcomes on core clinical indications against all competitors. PBS reference pricing provides this high reward for success in true competition on a level playing field. These policies, in retrospect, paid insufficient attention to supporting and developing PBS reference pricing as both a fiscal lever over patented drug prices and a means of developing a sustaining local pharmaceutical industry capable to responding to public health demands and encouraging employment amongst our best science and chemistry graduates.

### Australian Generics Policy and Reference Pricing After the AUSFTA

2004 was a pivotal year for Australian generics medicine policy. That date marked the signature and subsequent entry into force (on 1 January 2005) of the pharmaceuticals-related provisions of the *Australia-US Free Trade Agreement *(AUSFTA). Particularly important in this context was the potential for manufacturers of patented pharmaceuticals (through agencies such as an AUSFTA Medicines Working Group and carefully staged conferences on the future of the PBS) to lobby for or against Australian medicines policies, relying on the trade sanctions-backed Annex 2C.1 principles (for example, reward of pharmaceutical 'innovation') and article 17.10.4 of Chapter 17 (introducing so-called 'linkage evergreening,' as explained in another paper in this series). Since that time, it appears to have been very difficult for the Australian federal government to develop policies that would facilitate the growth of a high value-added generic pharmaceutical sector. It also seems to have been more difficult for government to resist the policy demands of the 'brand-name' drug industry, particularly those in relation to 'eliminating' reference pricing. Of even greater concern, is the possibility that the Australian government struck a covert deal during the AUSFTA negotiations (in contradiction of its public assurances), to subsequently remove PBS reference pricing. Such an allegation is controversial, nevertheless, is easily countered by continued government support for reference pricing as a valuable fiscal lever over the expanding costs of patented pharmaceuticals.

Supporting such an hypothesis was a Conference Agreement on the *Medicare Prescription Drug Improvement and Modernization Act *2003 (US) which obliged the United States Trade Representative (USTR), the Secretary of Commerce, and the Secretary of Health and Human Services to analyse:

whether bilateral or multilateral trade or other negotiations present an opportunity to address...price controls and other such practices and ...shall bear in mind the negotiating objective set forth in the *Bipartisan Trade Promotion Authority Act *of 2002 **to achieve the elimination of government measures such as price controls and reference pricing **which deny full market access for United States products. In so doing, the agencies shall provide periodic and timely briefings for the Committees of the House and Senate listed above, with an interim briefing no later than 90 days after enactment to address **negotiations to establish a U.S.-Australia Free Trade Agreement **and, as appropriate, other current negotiations [17]. [emphasis added]

Prior to signature of the AUSFTA, Australia's chief negotiator went on record before a Commonwealth Senate Select Committee on the AUSFTA, as stating that the agreement had merely preserved the status quo in areas such as patent terms, data protection, market entry after patent expiry, compulsory licensing, parallel importation and intellectual property harmonisation [18]. He admitted that article 17.10.4 did require legislative change by Australia, but disagreed with the submission of the Australian Generic Medicines Industry Association (GMiA), in denying those provisions were intended to promote 'evergreening' of brand name pharmaceutical patents. Most importantly, in relation to the PBS and the AUSFTA Annex 2C provisions he stated:

We went into these negotiations with an absolutely clear mandate to protect and preserve the fundamentals of the PBS. This is what this agreement does...**There is nothing in the commitments that we have entered into in Annex 2C or the exchange of letters on the PBS that requires legislative change **[19]. [emphasis added]

Australia's senior negotiator on the AUSFTA PBS provisions, likewise stated:

I would like to reinforce what Mr Deady has said, in that the [AUSFTA] PBS text-annex 2C and the associated exchange of letters-**entirely preserves the fundamentals of the listing and pricing mechanisms of the PBS**...The principles that are articulated in paragraph 1 of Annex 2C...do not convey any specific obligations on the parties and they are indeed consistent with the current principles and practices underlying the operation of the PBS...They do not prevent the continued priority being accorded to fundamental principles that are articulated in our national drug policy [National Medicines Policy], particularly in relation to affordable and timely universal access to medicines, innovative or otherwise. They do not preclude the continued recognition of the importance of public health as encompassed by Doha paragraph 6 [World Trade Organisation *Doha Declaration on TRIPS and Public Health*]...The text of the agreed principles in [Annex 2c.1(d)] states: *The need to recognise the value of innovative pharmaceuticals through the operation of competitive markets or by adopting or maintaining procedures that appropriately value the objectively demonstrated therapeutic significance of a pharmaceutical*. That text is specifically intended to reflect two systems...It was purely intended to reflect the US system in the reference to the competitive market, and in the reference to adopting and maintaining procedures that 'appropriately value the objectively demonstrated therapeutic significance,' it was intended to reflect the Australian system as it currently exists. The understanding was that the agreed principles were entirely consistent with the operation of the PBS in its current form and do not oblige us in any way to change the way in which we operate [20]. [emphasis added]

Yet, other evidence before the same Senate Committee expressed concern for the survival of the Australian generic pharmaceutical industry in the changed regulatory circumstances likely to arise as a result of the AUSFTA [21]. Valuing of 'innovation' as a policy principle was emphasised at the first meeting of the Medicines Working Group (MWG) in Washington DC in January 2006. Academic commentators argued, however, that 'valuing of innovation' was really an industry lobbying principle that needed to be far more thoroughly tested by democratic processes before it could be allowed to drive health policy in Australia. More public and stakeholder debate was needed on the definition of 'innovation' and how it should best be transparently and accountably measured. Extensive debate was also needed on its coherence with the existing principles of National Medicines Policy [22]. Certainly the Australian generic pharmaceutical industry was not as systematically consulted with and supported by the Australian AUSFTA negotiators, as was the multinational patented pharmaceutical industry by their US counterparts [23]. Many provisions in the AUSFTA, particularly those in Ch 17 introducing 'linkage evergreening,' extending patent terms for delayed marketing approval and restricting compulsory licensing, directly opposed the interests of Australian generics manufacturers and Australian public health. Of great concern would be whether the reference to valuing for 'innovation' in Annex 2C.1 of the AUSFTA was negotiating code for an agreement to facilitate the explicit agenda of US negotiators in breaking the PBS reference pricing link between innovative products and generic medicines.

On 1 August 2005, without consulting the Australian generic pharmaceutical industry (as it subsequently acknowledged), the government imposed a 12.5% mandatory cut in the benchmark price when a generic was first launched within a particular therapeutic class. It announced (in words that mirrored the those of Medicines Australia) it would like to achieve PBS savings by further cutting the profit margins of generic drugs, to allow 'headroom' for reimbursement of new, expensive, 'brand-name' (or 'innovative') drugs [24].

Article 2C.2(e) of the AUSFTA had required the selective promotion of transparency in Australian PBS listing processes (industry commercial-on-confidence claims were excepted). Annexes in trade agreements are designed apply to one party, rather than creating mutual obligations (although the principles of Annex 2C.1 explicitly create mutual obligations). This AUSFTA Annex 2C.2(e) obligation, however, was broadly in accord with Australia's National Medicines Policy [25]. In fact, it provided the impetus for the creation of the valuable Public Summary Documents (PSDs) providing community information on the outcomes of pharmaceutical company submissions from the July 2005 PBAC meeting. PSDs are likely to become valuable means whereby PBAC processes involving 'objectively demonstrated therapeutic significance' become recognized as the most efficient means of assessing the public value of innovative pharmaceuticals in a genuinely competitive market.

This start date for PSDs, however, conveniently post-dated Pfizer's successful immediate post-AUSFTA PBAC submission concerning its 'blockbuster' anti-cholesterol drug Lipitor (atorvastatin). Pfizer's application, in any event, was to change atorvastatin's PBS listing status from cost-minimisation (involving reference pricing because no clinical outcome advantage had been proven against the competitor simvastatin) to cost-effectiveness involving a price premium, so avoiding reference pricing and the generic 12.5% reduction. This was successful despite the fact that the PBAC noted that there were no head-to-head trials comparing atorvastatin against simvastatin, which measured hard outcomes like cardiovascular events, rather than surrogate markers such as changes in cholesterol levels [26]. Some members of the PBAC subsequently expressed concern about unusual departmental pressure concerning this decision [27]. In the author's opinion, the context and outcome supports the fact that the Lipitor decision had some connection with US AUSFTA negotiator demands that the Australian government trade off reference pricing, or begin to value innovation more through the vague standard of what they term the 'operation of competitive markets', although an equally valid hypothesis is that a variety of other industry lobbying forces were at work. The success of such pressure, should it be proven, if in any way influential in the ultimate decision, represents a serious beach of the integrity of PBAC processes and Australian health policy development.

After the AUSFTA, the Australian government propounded a variety of draft medicines-related policies for public and stakeholder discussion. These included: (1) Preferred prescribing to ensure that patients always have access to medicines at no more than the co-payment. (2) A two year phase-in for flow-on reference pricing changes for patented drugs that would allow sponsor companies to further demonstrate their cost-effectiveness. (3) Compensation to pharmacists for changes to pharmacy income flowing from the impact of policy change on the PBS. (4) Disclosure by manufacturers of the actual price at which drugs are sold. (5) A mandatory 5 per cent price cut for new generic medicines that are listed on the PBS (as well as the existing mandatory 12.5 per cent cut for the first new generic in any reference pricing group). These proposals were set in the context of continuing support for gradually strengthening the capacity of the PBAC cost-effectiveness process to assess the offered price for innovator drugs relative to both modeled or disclosed marginal cost of production and community value based on objectively demonstrated therapeutic significance in post-listing assessments [28].

In 2005–6, the Australian Federal Government joint Treasury, Industry and Finance Committee also developed draft policies which included a tender system for the supply of off-patent medicines to the PBS. This option involved the 'winning' manufacturer(s) being able to offer the drug to patients at a significantly discounted co-payment of up to 50%. It also required that PBS reimbursement accorded other drugs in the same therapeutic class be reduced to match the price offered by the winning tenderer, who alone could offer the drug at a reduced co-payment. A related proposal granted the winning tenderer six months of reduced co-payments against therapeutic comparators. At the end of this period, any manufacturer offering the reduced price would also be able to offer patients the discounted co-payment [29]. Estimated PBS savings resulting from these various proposals was between $300 and $800 million a year [30]. Yet, none of these policies was implemented and the suspicion is that they were merely bargaining chips.

Also in the mix were proposals drafted on behalf of patented, allegedly 'innovative' pharmaceutical manufacturers in Australia through their lobby organisation Medicines Australia. These involved limiting market competition the market place so that brand name pharmaceuticals could be listed in a category which did not allow them to be reference priced against generic competitors that were interchangeable at the clinical level [31]. One such suggestion allowed the PBS system to pay less for a generic medicine every time its sales increased by a set percentage. Others required generic manufacturers to compete for the right to a period of market exclusivity [32]. The most extreme policy position taken by Medicines Australia involved eventual replacement of the PBS with individual consumer medicines savings accounts [33]. The path to such an outcome will not be a short one but is likely to be paved with rubble from the dismantling of reference pricing and the resultant increasingly high and unsustainable PBS costs for patented pharmaceuticals, whose claims to innovation remain untested against evidence-based criteria of objectively demonstrated therapeutic significance against all market competitors.

The AUSFTA MWG met for the first time in Washington on 13 January 2006. At this inaugural meeting Australia's Trade Minister Mark Vaile stated:

"The core principle that we both agree on in this area...is recognising the value of innovation [34].

Documents about this AUSFTA MWG meeting were obtained under a Freedom of Information application [35]. They reveal that the first AUSFTA MWG meeting discussed only one Op-Ed and that included this statement: "Truly innovative cures should be referenced against innovation in other classes, rather than against generics."[36].

### Reference Pricing and the PBS F1-F2 Changes

Late in 2006, the Federal Minister for Health announced the final version of this round of PBS reforms. This endorsed what was, in substance, the Medicines Australia policy proposals for a new stand-alone (F1) classification in the PBS for non-clinical interchangeable medicines. The minister announced:

First of all, we are dividing drugs on the PBS into two categories – Formula 1 and Formula 2. Formula 1 drugs will essentially be drugs on patent, drugs for which there is a single brand. Formula 2 drugs are essentially drugs off patent, drugs for which there are multiple brands. **Now reference pricing arrangements, as they have traditionally operated...will not operate in F1**....[22] [emphasis added]

The *National Health Amendement (Pharmaceutical Benefits Scheme) Bill 2007 *proposes to add new sections 85AB, 85AC to the *National Health Act 1953 (Cth)*. These fracture the unitary PBS formulary into two: F1 for patented or allegedly 'innovative' medicines and F2 for generic medicines. Price cuts and disclosures will be imposed *only *on F2 generic medicines (new Division 3A of Part VII). Reference pricing will be limited to a few existing F1 therapeutic groups, or to where they have been established on the imprecise standard that comparitors are 'interchangeable on an individual patient basis' (proposed sections 84 AG and 101 (3BA)). There will be no ongoing reference price links between medicines listed on F1 and those listed on F2 [37].

The new F1 category represented a fundamental challenge to the integrity of PBS reference pricing. The extent to which the inability to 'reference' the price of F1 to F2 drugs and the precondition of 'interchangeable on an individual patient basis' actually undermines reference pricing in the future remains to be determined. Perhaps the latter standard can be made the subject of criteria more in accord with the Australian mechanims of objectively demonstrated therapeutic significance. If increasing numbers of patented pharmaceutical manufacturers are successful in obtaining PBS F1 listing for pharmaceuticals that involve only minor molecular alterations ('me-too' medicines), on the basis of placebo controlled trials, that will indeed signal that reference pricing has been traded away. Another test of the survivability of reference pricing will be the number of drugs that move from F1 to F2 categories as 'incremental innovation' occurs in competitor molecules and promised health outcomes, price determinations and classifications are reassessed by the PBAC. What eventually happens to Pfizer's 'blockbuster' lipitor (atorvastatin) may become a litmus test of whether the PBS system, as a result of such changes, has become more responsive to multinational corporate lobbying based on valuing innovation by (distorted) market forces, rather than the 'cost' and 'responsibility' factors by which the National Medicines Policy orients industry renewal towards the public health needs of Australian citizens.

The F2 category of medicines where there are many brands listed and groups of medicines that are interchangeable between patients and pharmacists offer price discounts to suppliers. From 1 August 2008 a price drop of 2 per cent a year will occur over three years for F2A medicines where price competition between brands is low. A one-off price drop of 25 per cent will occur for medicines categorised as F2T where price competition between brands is high. For a defined list of medicines, this will be phased in over remaining patent life. Given examples of F2T medicines are simvastatin, omeprazole, ranitidine, amoxycillin and felodipine and around 100 other molecules currently costing the PBS $2 billion a year. It has been suggested that angiotensin II receptor inhibitors will not be included, though it remains unclear whether this can be interpreted as *ad hoc *policy at the behest of industry lobbying. Over time, the Government plans to reimburse only the actual price at which the F2T medicine is being sold [38]. Pharmacists have been promised compensation of 40 cents for every script processed through OBS Online from July 1 2007 and national full line wholesalers $69 million over three years to the Community Services Obligation Funding Pool.

It is difficult to see how these new F1 and F2 categories fulfil any of the basic criteria of the National Medicines Policy or are in the long term interests of Australian public health. The new F1 category clearly only benefits multinational manufacturers of new patented medicines and makes it difficult for their claims to innovation to be tested on criteria of objectively demonstrated therapeutic significance. The F1 PBS category is presumptively anti-competitive and pro-monopolistic in that it shields products from having to prove their claims for PBS reimbursement against competitors. Its continuance should be thoroughly investigated by Australian market competition and anti-trust regulators.

It will be critical for the practical survival of reference pricing under the PBS, that new patented pharmaceuticals continue to be listed in price groups classified (in accord with s 101(3) of the *National Health Act 1953 (Cth)*) on the basis of hard therapeutic outcomes (QALYS, for example from blood pressure, cholesterol or gastric ulcer reduction). The F1-F2 category, in other words, is fundamentally unsuited to allowing the PBS system to remain responsive (in terms of equity of access and moderation in public health expenditure) to expensive new generation biologic generics and nanopharmaceuticals. It is to be hoped that a full review of the F1-F2 changes takes place with a view to their dismantling.

The Australian health minister admitted these F1-F2 changes did not greatly advance the interests of either generic manufacturers or pharmacists, which, of course, is almost an admission that they served chiefly the interest of patented multinational pharmaceutical manufacturers, represented by Medicines Australia.

The Generics Medicine Industry Association [GMiA] is not, as I understand it, especially happy with these changes... [but]... we are, as part of these changes, ruling out a tendering system and I think that the whole sector, including GMiA, should be pleased that we are not going down the New Zealand path. The final point I would make is that by removing the gross discounts from the system, we should ensure that domestic generic manufacturers are less at risk from predatory newcomers such as some of the Indian generic drug manufacturers [39].

Saying that the GMiA was unhappy with the proposals was an understatement. The GMiA chairman John Montgomery stated:

The reforms dismantle reference pricing, encourage 'evergreening' and provide no incentive for the use of true generics. This flies in the face of the government's stated aim of ensuring sustainability of the PBS...Eliminating the ongoing [reference] price link between patented medicines and medicines producing the same outcome but no longer patent protected means Australian taxpayers will be paying higher prices for essentially the same health outcome...The reforms...will undermine [PBS] fundamentals and will paradoxically increase its costs [40].

These creation of an F1 PBS category largely conforms to plans of the multinational brand name pharmaceutical manufacturers and US AUSFTA negotiators to undermine reference pricing. For this reason the interest of US representatives in the new F1-F2 reforms manifested in the second meeting of the AUSFTA MWG in early May 2007, may be revealing [41]. The F1-F2 changes, however, are most problematic for being merely the most recent in a list of sporadic Government interventions, over the past twenty years, designed to support the Australian pharmaceutical industry, but failing to deliver industry renewal comparable to that achieved as diversely comparable (to pick but a few examples) as Ireland, Sweden, Canada or India (as highlighted in the latter case, by Lofgren's article in this issue). The temporary rejection of close-bid competitive tendering, for example is curious. Such a tendering system may actually have favoured a generic medicines industry in Australia and certainly facilitated lower prices closer to the marginal cost of production [42]. Tendering under the PBS is certainly a policy worthy of reconsideration in policies designed to facilitate industry renewal in accordance with the National Medicines Policy.

### The Australian Generics Medicines Industry and Other Recent Reforms

On the positive side, under amendments to the *Patents Act 1990 (Cth) *passed on 14 September 2006, a relatively narrow 'springboarding' exemption (which applied only to extended term of a patent for an active pharmaceutical ingredient (API)) was replaced with a general exemption to infringement of a 'brand-name' pharmaceutical patent (defined broadly as including APIs, methods, uses and products), for a generic seeking to get access to originator data to develop a product prior to placing it on the market after originator patent expiry. This 'springboarding' amendment did not apply to medical devices (which as we shall see, may be a significant oversight in relation to nanotherapeutics). The Australian generic pharmaceutical industry will undoubtedly gain some assistance for this change.

As at early 2007, the Australian generic manufacturing industry remained comparatively small (employing approximately 3,000 people) and characterized increasingly by cross ownership and licensing arrangements with multinational brand name companies [43]. One of the largest manufacturers (65% of the generics market) was Alphapharm (parent company Merck KgaA). Shortly after announcement of the generics-adverse 2006 PBS F1-F2 reforms, Alphapharm began negotiations for a sale of its generics business to the Indian company Ranbaxy [44]. Eventually Alphapharm was purchased by the US generics company Mylan Laboratories. Sigma Company, the next largest generics manufacturer, merged in 2005 with Arrow Pharmaceuticals, to form Sigma Pharmaceuticals [45]. Early in 2007 The US Federal Trade Commission (FTC) approved Hospira's US$2 billion takeover of the Australian generics injectables manufacturer Mayne Pharma on condition Mayne sold the rights to certain analgesics and an iron chelating agent, to US Barr Pharmaceuticals. Genepharm Australasia, one of the most forward thinking local generics manufacturers, recently acquired the Australian operations of the New Zealand-owned Douglas Pharmaceuticals. Generic Health is also an important local generic manufacturer with a progressive focus. Hexal Australia is now part of Sandoz, the generics arm of the Novartis Group, following Novartis' acquisition of Hexal's parent company, Germany-based Hexal AG. In 2006 PharmAust announced it would be importing generic medicines sourced in Malaysia by Xepa-Soul Pattison, a division of Apex Healthcare [46].

Another feature of the Australian generic pharmaceutical industry is that less than 5 percent of sales is represented by products made from locally manufactured active pharmaceutical ingredients (APIs), notably those derived from alkaloid extraction based on poppy farming in Tasmania (operated by GlaxoSmithKline and Johnson and Johnson) and the small scale facilities of the Australian Nuclear Science and Technology Organization and the Institute of Drug Technology. Fully imported products or fully finished products packaged locally represent about 62 percent of sales [47].

Thus, to the limited extent that any pharmaceutical manufacturing is undertaken in Australia, both the generics and the international brand companies operate relatively small scale and low-tech operations. Adding to competition problems for the Australian generic pharmaceutical industry is the proliferation of linkages (licensing, co-marketing or distribution agreements etc) between originator and generics firms, particularly in the form of so-called 'authorised' generics, 'pseudo' generics, or 'fighting brands' [48]. These are not generics in the usual sense of this term, but simply repackaged brand name products designed to 'warn-off' competition from 'blockbusters' whose core product patent is about to expire [49].

The extent to which government policy (or its absence) has contributed to the relatively subdued state of the current Australian generics pharmaceuticals industry, is unclear. Certainly, however, such policy has not involved forward planning, coherent with all elements of the National Medicines Policy, to assist the Australian nano/bio sector meet impending challenges such as those next discussed.

### Industrial Renewal: Biologic Generics

It is estimated that several hundred new 'biologic' drugs are now in development pipelines. These include, for example, growth hormone, insulin, granulocyte-macrophage colony-stimulating factor (GM-CSF), or erythropoietin. Such drugs are distinctively derived from living cells and their manufacturing companies often prefer to call themselves 'discovery generics', to highlight the amount of innovative research required for successful product development of these generic products. The current worldwide market for protein-based biotech. drugs, is over $20 billion. Biotechnology patents increased substantially in most nations in the period 1991–2002, including Australia (19 to 100), Canada (53–136), Sweden (24 to 93), US (1160 to 2342) and EU (650 to 2025). India (3 to 28), China (0 to 49) and Ireland (6 to 7) increased by comparatively small amounts, but achieved the strongest gains in the most recent years [50].

In the bio/nanopharma sector, Australia retains a leading role in the Asia-Pacific region and ranks number sixth the world in terms of number of firms [51]. Without careful policy attention this positive situation may not continue. Remove Australia's three largest biotech companies (CSL, Cochlear and ResMed), for example, and the sector as a whole suffered a 14.6% decline of share price in 2006 (the NASDAQ Biotech Index falling 14.3 per cent in the same period).

One main obstacle to generic investment in such biologics, is the difficulty in obtaining regulatory quality, safety and efficacy approval for marketing. To achieve such marketing approval, a generic 'biologic' manufacturer must uniquely prove to a regulator use of the same protein expression system, purification protocol, and delivery technology as in the original patent. Unusually stringent aseptic production techniques are required to guard against contamination.

Safety, quality and efficacy regulators also consider that there are significant unresolved scientific issues about how to establish bio-equivalence between complex biological macromolecules. A protein, for example, can be folded, glycosylated, and methylated in quite different ways if expressed in mammalian or bacterial cultures. Likewise, a generic monoclonal antibody may bind to the same antigen, but through an alternate binding site and with an altered affinity from the original antibody. All of this may alter a product's pharmacokinetics and pharmacodynamics from the brand name competitor. The source material in biologic manufacturing is likewise not as readily classified as involving chemicals or standard generic pharmaceutical active product ingredients.

Most medical ethics guidelines preclude clinical trials on a product that is demonstrably inferior to the current standard of care. Under current regulations, as long as a company can continue making medically significant improvements on a therapeutic protein, it may be able to retain an exclusive market indefinitely without having to repeat full-scale clinical trials. Amgen appears to have used that approach in developing an improved version of its blockbuster treatment for anaemia, Epogen (Aranesp). In Europe, the Schering company likewise has gained approval for a version of interferon-alpha called PEG-interferon alpha, in which a polyethylene glycol (PEG) moiety increases the half-life of the protein in the body, reducing dosing frequency.

Similarly, new generic production facilities often generate biologics with increased purity from the original, placing pressure on 'discovery generic' manufacturers to perform additional clinical trials. These, through inconvenient and a substantial additional expense, are likely to be less risky to patients, however, than the original studies, because the underlying principle of the drug's action has already been proven and the clinical end point is known. Some 'biologic' manufacturers have even filed for a new patent after significantly altering the production process. Eli Lilly, for example, developed a new manufacturing process for its human growth hormone and had the protein approved as a new orphan drug (Humatrope). Overlapping product patents, process patents, use patents and purity patents are likely to spur litigation for product exclusivity in this area [52].

Such regulatory problems contributed to the fact that, in 2004, 2005 and 2006, only 5, 2 and 4 biopharmaceuticals respectively, were transferred from the US Center for Biologics Evaluation and Research to the Center for Drug Evaluation and Research for successful biologics license applications [53]. A proposed US Federal Access to *Life-Savings Drugs Act *is intended to alleviate such problems. It allows abbreviated approval of biological products that share the "principal molecular structural features" of previously approved brand-name products. Approval for pharmacy substitution is conditional on regulators approving a biologic as a clinically "interchangeable" product, rather than a "follow-on" (or "me-too'). The Bill grants the secretary of the Department of Health and Human Services (DHHS) the extraordinary discretion (and responsibility) of determining on a case-by-case basis, whether additional clinical trials are required [54].

Yet, in 2006, the European Medicines Agency (EMA) following new guidelines, recommended approval of Sandoz's Omnitrope, a generic version of an existing growth hormone pharmaceutical. The EMA, unlike the US Food and Drug Administration (FDA) has guidelines assisting generic manufacturers wishing to market 'biogenerics' Further, companies such as Momenta Pharmaceuticals are utilising technologies that analyze the structure of complicated sugar molecules and possibly proteins, smoothing regulatory safety, quality and efficacy approval of these replicant pharmaceuticals [55].

Certain geographic and political areas are racing ahead with biologic development. In Denmark, for example, strengths in clinical science base, management and established indigenous pharmaceutical companies are supported by policies facilitating start up and collaborations (for example Novo Nordisk and Leo Pharma (diabetes) and Lundbeck (psychiatric and neurological disorders)). A particular element in Scandinavian success in this area may be 'Medicon Valley', (around Copenhagen and Malmö in Sweden), which, along with Cambridge in the UK and Basel, is one of Europe's top three biotech clusters [56].

Australian pharmaceutical policy makers need to learn the lessons of the industry renewal policies that have been applied, or are being attempted, to achieve such results with biologic generics. Breaking the reference pricing linkage between 'innovative' and 'generic' drugs may not be useful in this context.

### Industrial Renewal: Pharmacogenetics

Another biopharma area where carefully organized policies, building on existing skills and facility strengths, could promote Australian industrial renewal, is pharmacogenetics (the science of studying genetically-determined responses to medicinal drugs). Based on recent UK and US studies, about 1 in 15 admissions to Australian hospitals are due to or involve adverse drug reactions, many of these directly leading to adverse health outcomes [57]. Such harmful side effects vary between individuals and range from failure to respond therapeutically, to minor illness and even death [58]. A few Australian companies are already starting to invest in this area. One prominent example is Genetic Technologies Ltd, which is licensed by Myriad Genetics (USA) to carrying out BRCA breast cancer genetic screening. Australia, generally, has a strong related skills base in genetic sequencing.

Predicted developments in pharmacogenetics include (1) recording of individual patient pharmacogenetic profiles (2) establishment of prescribing guidelines, that will relate dose to genotype and highlight the possibility of adverse drug interactions (3) development of new drugs for patients with specific genotypes (drug stratification). This latter area could be of particular policy value in the context of Australian biopharma industry renewal. Pharmaceutical industry interest may extend to 'packaging' drugs along with genetic tests and takeovers or licensing of genetic test manufacturers [59].

The US FDA's approval of the AmpliChip CYP450 (Roche and Affymetrix) for *in vitro *diagnostics represented a significant regulatory advance for pharmacogenomics Yet, as with 'biologicals,' regulatory changes necessary to facilitate uptake of (and public benefit from) such pharmacogenetic developments have yet to be systematically considered by Australian health policy makers.

Privacy laws, for example, will need to mesh with the capacity of a simple finger prick, mouth wash, or hair sample to obtain genetic information enabling a doctor to rapidly determine the likelihood of a drug's efficacy and side effects. If pharmacogenetics is to minimize drug expenditure by reducing wastage and simplify post-marketing surveillance, then both Therapeutic Goods Administration (TGA) and the PBS officials will need to be actively involved in policy development. Under definitions of reference pricing prior to the F1-F2 categories, for example, new patented drugs seeking PBS listing in conjunction with a genetic test would still need to be evaluated for comparative cost-effectiveness against existing marketed products (without linked genetic tests). Clinical trials are becoming increasingly expensive and pharmacogenetics could provide a seemingly attractive way of reducing industry dependence on them for regulatory approvals and post-marketing surveillance. The Novartis Institutes of Biomedical Research has recently been promoting use of biomarkers to select research subjects with the idea of improving the efficiency of pharmaceutical clinical trials. Despite cautious present investor interest, linking medicines with a genetic test could facilitate valuable long term diversification in the Australian bio/nanopharma industry.

### Industrial Renewal: Nanotherapeutics

Medical nanotechnology involves the development of drug/invasive therapeutic device products controllable at atomic, molecular or macromolecular levels of approximately 1–100 nanometers. Nanostructures have much greater strength, stability and surface area per unit mass than standard materials and those below 10 nm possess quantum effects where size may control, for example, the specific wavelength of emitted light [60].

Nanotechnology is a rapidly expanding area of medical research and development globally.[61] Over 200 companies are actively involved in this area, viewing nanotechnology is having a powerful enabling function that enhances the effectiveness and market competitiveness of existing health technologies [62]. Peptide nanotubes, for example, have been investigated as the next generation of antibiotics [63] and as immune modulators [64] Nanomedical applications been investigated in neurosurgery [65], cardiac surgery [66] and blood disorders [67] Most major pharmaceutical companies have substantial investments in nanotechnology [68].

In Australia, nanomedicine is a rapidly growing industry sector. Nanotechnology is a priority area for Australian Research Council (ARC) funded research (A$53,013,909 in 2002–03), many collaborations being promoted by the ARC Nanotechnology Network (ARCNN) [69]. Starpharma, for example, (with US-based Dendritic NanoTechnologies) and Australian government and US National Institutes of Health (NIH) funding, is developing VivaGel™ as an HIV-prevention dendrimer-based microbicide gel. VivaGel™ represents bottom up nanotechnology and involves a well-defined synthetic polymer, made by adding monomers in a branching manner, binding to glycoproteins on the surface of HIV and thus preventing, in a dose-response manner, HIV binding to receptors on T-cells. VivaGel™ is the world's first dendrimer-based drug to be approved for human trials by US FDA (phase 1 study completed 2004). pSividia has developed Brachysil™ a nanostructural, porosified, biosilicon platform technology for controlled drug delivery and already have a licensing agreement for it with a US company based in China.

At present, however, most regulatory concern in Australia seems to be focused generally on the safety of nanotechnology, rather than on facilitating venture capital for a nanomedicine industry systematically focused, through good regulatory architecture, on public health outcomes. A major concern is that highly reactive and mobile engineered nanoparticles (ENPs) may present unique health risks when used in medical applications [70]. There are currently no effective methods to monitor ENP exposure risks [71] Research suggests that the health risks of nanostructures cannot be predicted *a priori *from their bulk equivalent. In animal studies, short term exposure to ENP's has produced dose-dependent inflammatory responses and pulmonary fibrosis. Some engineered nanoparticles have also been shown to preferentially accumulate in mitochondria and inhibit function, others may become unstable in biological settings and release elemental metals [72].

Despite such findings, the US FDA appears to have assumed that macroscale safety may translate to that at the nano level [73]. A nanoparticulate reformulation of an existing drug, for example, has been deemed by the FDA not to require an Abbreviated New Drug Application (ANDA) because bioequivalence was established [74].

These developments suggest that the Australian government should take a stronger long term policy interest in public benefit-focused industry renewal in the nanotherapeutics sector. A recent Senate Inquiry recommended creation of a working party to consider creation of a distinct, permanent regulatory body for nanotechnology [75]. The latter approach was taken with gene technology under the *Gene Technology Act *2000 (Cth) [76]. Such a broad licensing approach, encompassing regulatory industrial, agricultural and therapeutic applications may not be the best vehicle for encouraging renewal in the uniquely complex Australian bio/nanopharma sector.

Appropriate regulatory changes could favour the development of a biopharma industry where existing off-patent products are re-badged to become more profitable with a more effective nano-based delivery system. On the other hand, hasty regulatory approval of nano-versions of existing drugs (as is the case with generic 'biologicals') could place expenditure burdens of public health systems and risk damage to public health. In this context, given the presumptive claims that nanomedicine manufacturers will make for reimbursement reward of their 'innovation', the maintenance of a robust system of PBS reference pricing will be critical to ensuring that the Australian public obtains value for its nanomedicine expenditure. A recent European Science Foundation report recommends that the flexible enabling functions of nanotechnology in medical applications may be lost if coordinated policies facilitating investment and efficient regulation are not developed [77]. One of the best models for facilitating community value from nanotechnology research may be the Nanotechnology Victoria (Nanovic) consortium (Universities of Melbourne, Swinburne and RMIT with the CSIRO) receiving start-up funding from the Victorian Government [78].

### Sustainable Industrial Renewal and Global Public Goods

Global public goods (increasingly defined in global regulatory debate in a broader sense than under traditional economic theory) provide benefits from which no individual should be excluded. They span national, cultural and generational boundaries, their consumption theoretically not creating rivalry. Some are 'merit goods' in the sense that their promotion and protection supports values such as justice and equity, as well as international human rights, and requires that contrary influences emerging from pro-monopolistic markets be overridden. Examples of global public goods include not only clean air, peaceful societies, control of communicable disease, transport and law and order infrastructure, but systems of universal (taxpayer-funded) health care and pharmaceutical research and development oriented by percentage GDP prize funds towards the global burden of illness. Some of these require international co-operation for their production (particularly safety and cost-effectiveness evaluation of new health technologies). Emphasis upon such goods highlights the need to seriously consider sustainability in the regulatory architecture of markets.

Affordable access to essential medicines is increasingly recognised as a global public good, providing an essential precondition to a reasonable quality of life for a significant proportion of every human population, being systematically underprovided by private market forces and imposing burdensome international externality costs on third parties [79]. Further, affordable access to essential medicines appears to be emerging, both academically and in practise, as a core part of the international right to health in article 12 of the *International Covenant on Economic, Cultural and Social Rights *(article 25 of the *Universal Declaration of Human Rights*). One recent manifestation was the *Doha Declaration*, which affirmed the capacity of WTO members to use to the full exceptions in the Trade Related Intellectual Property Rights agreement ("TRIPS") to promote public health by facilitating access to affordable medicines [80]. It is also specifically referred to in article 14 of the UNESCO *Universal Declaration on Bioethics and Human Rights *[81].

Global public goods, both in general and as directly related to the bio/nanopharma sector, in the immediate future, will have to be financed at the national level, with sector specific incentives. This will probably remain the case, at least until universal threats such as bioterrorism, emergent infectious disease and climate change, create sufficient political will for reform of world governance structures This may particularly be so if the claims of innovation by such technologies are undermined by their largely unaccountable expense [82]. Safety and cost-effectiveness evaluation of health technologies is one global public good that would benefit greatly from improved financial and administrative co-operation between nations, linked with enhanced democratic involvement [83]. Reference pricing provides a crucial mechanism in this context, by encouraging a market focus on objectively demonstrated improvements in health outcomes. Fortunately, Australia's National Medicines Policy has already established aims broadly in accord with this public goods focus on industrial renewal.

One specific strategy is for public goods-focused researchers to incrementally innovate patented drug molecules and, by institutionally treating the new patent as a public good, make the product available cheaply to poor patients in the developing world. This is an approach that if supported by appropriate policy, might greatly enhance the commercial prospects of companies in the Australia bio/nanopharma sector.

English medical researchers for example, recently redesigned an effective but extremely expensive Roche drug for hepatitis C, called pegylated interferon, so that its large sugar molecule (which increased its half life) on the inside, rather than the outside. The Shantha corporation in Hyderabad, which had made the world's first cost-effective hepatitis B vaccine (and was already making the original interferon), agreed to make the new medicine with the Indian government subsidising the necessary pre regulatory approval clinical trials. The UK scientists are also working on an innovative 'ethical pharmaceutical' for visceral leishmaniasis (kala-azar), a fatal disease in Brazil, Bangladesh, India and the Sudan transmitted by sandflies [84].

Industrial renewal in the Australian pharmaceutical industry will not occur without robust competition between manufacturers and ingredient suppliers in a regulatory environment that favours public goods. This being so, the lack of interest shown by the existing Australian generic pharmaceutical sector in the China-Australia Free Trade Agreement is remarkable.

China is one of the world's largest manufacturers of generic pharmaceuticals. In 2001, the sales income of China's (largely generic) pharmaceutical industry totalled US$21 billion [85]. China's pharmaceutical market averages 18–20% growth over the last twenty years, significantly higher than US and European growth over the same period. By 2020, China will have the world's largest pharmaceutical market.

The Australia-China Trade and Economic Framework was signed during the visit of Chinese President Hu Jintao in October 2003. This Framework sets the direction for the trade and economic relationship in the long term and included a commitment to conclude a Free Trade Agreement feasibility study by 31 October 2005. Its purpose is to enhance trade, investment and economic cooperation and build on Australia's commercial relations with China in a number of key sectors. It also commits the parties to further trade liberalisation.

One of the most common models for pharmaceutical development in China involves joint ventures with local partners facilitating regulatory approval and market share. China currently produces over 1,350 medicines in 24 classes. Almost all these are what may be described as "generic" drugs. In recent years, China has patented only two brand name or 'innovative' drugs (arteannuin and sodium dimercaptosuccinate) that have received international marketing approval. Yet China has strong ambitions in the innovative drug field, being hampered only by a present lack of access to drug design and regulatory expertise such as that possessed to a level of international excellence by Australia.

China acceded to the WTO on December 11 2001. In doing so, China agreed to restructure its domestic legal system to, amongst other things, comply with the obligations of the WTO TRIPS agreement. Late in 2002, a year after its accession to the WTO and agreement to abide by TRIPS, China passed its *Measures for the Administration of Pharmaceutical Registration (for Trial Implementation*) and *Implementing Regulations for the Law of the People's republic of China for the Administration of Pharmaceuticals*. Under these laws, once a pharmaceutical has been approved for domestic production the State Food and Drug Administration (SFDA) will not permit other companies to produce or import it for "monitoring periods" of 3–5 years. The chief purpose of these "monitoring periods" is to check for side effects, but of course it also accords a valuable period of market exclusivity. A 'generic' manufacturer seeking market entry makes an application to provincial drug authorities, who arrange on-site testing of samples. The SFDA will then conduct a comprehensive review, before deciding whether to issue a Pharmaceutical Production Permit. The procedure is similar for the issuing of a Pharmaceutical Processing and Export Approval Document.

When interviewed as part of an Australian Research Council grant in 2005, senior representatives of the generic pharmaceutical industry in Australia appeared concerned to deny market access to Chinese products, ostensibly on safety and quality grounds. This cannot be a sustainable regulatory ambition, as the Chinese generic industry develops and is unlikely to be in the long term national interest of the Australian public. China is emerging as a great potential market for Australian biotech and nanotech pharmaceuticals. pSivida Ltd, for example, an Australian listed public company with a substantial shareholding in pSiMedica Ltd (UK), has patented in China its nanotech silicon drug delivery system (BioSilicon™). The China-Australia Free Trade Agreement offers Australia an opportunity to share its pre-eminent regulatory strengths in safety, quality, efficacy (through the TGA) and cost -effectiveness analysis (via the PBAC) with China, in return for enhanced access for its bio/nanopharma products to the Chinese market. In time this could become a stepping stone to a multilateral treaty on safety and cost-effectiveness assessment of new health technologies [86]. This background makes it remarkable then that discussion about generic pharmaceuticals appears not even to have been raised as a topic of discussion in negotiations related to the China-Australia Free Trade Agreement [87].

Recent obligations acquired under the Australia-US Free Trade Agreement (AUSTFA) may play a problematic role in the process of industrial renewal in the Australian bio/nanopharma sector. So-called 'Fast Track' provisions require discussion between the US FDA and the Australian TGA about making "innovative" pharmaceutical products more speedily available (Annex 2C.4). It remains controversial whether these will be carved out from the New Zealand obligations under the proposed Australia-New Zealand Therapeutic Products Agency [88]. There are also obligations acquired under the AUSTFA requiring 'valuing' of pharmaceutical "innovation" through either the operation of "competitive markets" (the US position, requiring an enhanced role for competition and anti-trust regulators) or "objectively demonstrated therapeutic significance" (the Australian position, supporting an enhanced role for the PBAC in ensuring that the PBS expenditure on new PBS-listed products is commensurate with evidence-based assessments of their comparative community value and cost of development) (Annex 2C.1)[89]. The "generic" (rather than "innovative") status of biologic and nanoparticulate versions of off-patent drugs will be particularly difficult to assess [90].

South Korean negotiators of the Korea-US Free Trade Agreement (KORUSFTA) appear to have taken a much stronger stance in favour of their emerging biologic generics industry [91]. The South Korean government was so impressed by the socially and scientifically sound economic incentives offered by Australian PBS evidence-based cost-effectiveness and reference pricing system, that article 5.2 of the KORUSFTA permitted South Korea to establish a PBS-like reimbursement system for pharmaceuticals or medical devices where the amount paid was not based on 'competitive market-derived prices'. That article indicated that if it did so, then amongst other things, it had to 'appropriately recognise the value of patented pharmaceutical products' (article 5.2 (b) (i)). Article 5.1 (c) and (e) respectively echoed PBAC-type evidence-based pharmcoeconomic analysis by referring to 'sound economic incentives' as a method of facilitating access to patented medicines and 'transparent and accountable' procedures as a means of promoting innovation [92].

A major report in the UK has also recommended the PBS evidence-based cost-effectiveness system linked to a central government price negotiation as a model for that nation [93]. Interest is gathering in the US for a similar linkage of health technology cost effectiveness analysis with central government price negotiation [94].

Biosecurity is also an important global public good with important connections to the biopharma sector. At the time of the anthrax biosecurity threat in the US, it became clear that protection of the population in many developed nations may be crucially dependent on the capacity of generic manufacturers to rapidly increase and stockpile necessary pharmaceuticals, if necessary under a compulsory license (Thailand, for example, recently confirmed it would issue compulsory licences permitted under the WTO TRIPS agreement, to buy generic versions of Sanofi-Aventis' anti-blood clotting pharmaceutical Plavix and Abbott's anti-HIV/AIDS medicine Kaletra).

As the Australian pharmaceutical industry increasingly researches biologic products, these may (rarely) produce unintended outcomes that create biosecurity implications. An example is the unintended development of a highly virulent and dangerous mousepox IL-4 [95] or botulinum in milk [96].

In 2005, scientists from the US Armed Forces Institute of Pathology published the full sequence of the highly virulent strain of influenza virus that caused the Spanish influenza pandemic in the winter of 1918–1919 and killed up to 50 million people worldwide [97]. Further work based on the sequence led to the synthesis of an influenza strain which showed a high virulence and mortality rate when tested in mice [98]. Such developments confirm that recent developments in genetics, genomics and other areas of pharmaceutical development might (intentionally or unintentionally) create biosecurity hazards [99]. A National Centre for Biosecurity (NCB) has been established at the Australian National University to coordinate scientific, legal and policy expertise in this area.

The capacity of Australia to respond to a natural disaster or bioterrorist threat may crucially depend on our capacity to increase supply of blood and blood products. The on-shore capacity of the Commonwealth Serum laboratories (CSL) Pty Ltd is likely to play a crucial role in such a rapid response situation. Continuance of the CSL monopoly in this area has been challenged by an AUSFTA side-letter that creates a mechanism for opening up the area for competitive tender [100]. It will also depend upon Australia possessing a sophisticated local generic manufacturing capacity capable of responding to a compulsory license in a national emergency.

The Commonwealth Government Review of Australia's Plasma Fractionation Arrangements, after an exhaustive study of European and US arrangements, concluded that overseas fractionation of Australian plasma would involve significant transitional costs ($A75million) and because of yield considerations, there would be the potential for an ongoing shortfall in the supply of IVIg and other plasma derived products. The Review also found major potential supply chain risks in overseas fractionation. The Review recommended that the Federal government maintain the reservation exempting plasma fractionation services from the government procurement provisions of Chapter 15 of the AUSFTA. It recommended that the Federal government support CSL Bioplasma in this area in part because of the crucial role that company could play in Australia's biotech industry renewal [101]. In coming to such conclusions the review undoubtedly assisted to focus industrial renewal in this sector not only on positive public health outcomes, but public goods-supporting processes.

## Conclusion

The potential strengths of the Australian bio/nanopharma sector may be summarised as: (1) high levels of skills and expertise in basic medical research and healthcare in Australia. (2) excellent clinical and medical training programs and hospital/health infrastructure, well integrated with basic medical R&D institutes. (3) strong continuing support by Government for medical research through the National Health and Medical Research Council (NH&MRC). (4) high levels of expertise in pharmaceutical regulation (5) excellent capability in critical new knowledge areas and platform technologies: biologics, genomics, bio-informatics, nanotechnology and fast screening [102]. Australia possesses at least three centres with expertise in conducting Phase I pharmaceutical clinical trials [103].

This article has examined the proposition that Australian medicines policy has not been sufficiently supportive of a high value-added generics industry, or mechanisms facilitating that such as PBS reference pricing. It has argued that such policy has not involved a systematic plan for sustainable industrial renewal of the Australian bio/nanopharma sector in the context of the challenges such as 'biologics,' nanotherapeutics, pharmacogenetics, more globalised health technology regulatory assessment and biosecurity. It has shown made a reasonable case that the principles of the National Medicines Policy have not been consistently applied in this context and that the integration therein of extra-patent reward of innovation needs to be more systematically evaluated.

There is presently a critical window to shape the regulatory architecture for sustainable industrial renewal in the Australian bio/nanopharma industry. Australia for the time being, though having some cost disadvantages relative to comparable economies, has several non-cost advantages particularly in its skills base and safety and cost-effectiveness regulatory systems. The crucial policy task, over the next decade, may be to identify the critical high-efficiency niche bio/nanotech. production areas that should be consistently and systematically encouraged to meet the specific long term goals of the Australian people. In this respect, the venture capital evolution of a public-consortia model such as 'Nanovic' may present significant advantages. So too may, the retention and strengthening of PBS reference pricing as an important fiscal lever that promotes genuine and sustainable market competition, as well as Australia's promotion in international fora of a health technology safety and cost-effectiveness assessment treaty. It cannot be assumed that the long term interests of all Australian citizens and the goals of multinational corporate interests in the presently constituted patented pharmaceutical sector, will long coincide.
